# Efficacy of Additives to Morphine Pumps in Post-Operative Pain Control of Addicted Patients

**DOI:** 10.5812/kowsar.22287523.2046

**Published:** 2011-09-26

**Authors:** Sumitra G. Bakshi

**Affiliations:** 1 Department of Anesthesia, Critical Care and Pain, Tata Memorial Hospital, Mumbai, India

**Keywords:** Postoperative periods, Morphine, Pain, Patient-controlled analgesia

*Dear Editor*,

Acute pain management in opioid dependent patients remains a challenging and complex problem and it is becoming more common (1). In the acute pain setting, in addition to their daily opioid maintenance these patients need a multimodal approach (1). Imani et al. in their study have shown that instead of simply increasing the dosage of morphine, using morphine in addition to chlorpromazine, promethazine, midazolam and clonidine significantly controlled pain scores and increased patient satisfaction without having notable side effects (2). The results show that the mean pain scores are lower in the morphine plus protocol plus clonidine group versus the morphine plus protocol and morphine groups. Higher percentage of patients who were satisfied and lesser requirement of additional opioid were seen in the morphine plus protocol plus clonidine group. The total opioid consumption was higher in the plain morphine group, this can however be attributed to the study design as the morphine group was started on a higher basal infusion of morphine 40-mg over 20 hours versus 20 mg in the other two groups. The assumption that had the basal rate of morphine been reduced, these patients might have needed more extra opioid boluses may not essentially be true. These results raise a question as to whether addition of chlorpromazine, promethazine, midazolam are really worthy.

Another important issue that needs to be addressed is the compatibility of parenteral drug solutions. The decision to mix drugs should not be made without knowledge of their compatibility (3). Incompatibility problems are more likely to arise when small concentrated volumes are mixed in a syringe rather than in the large volume of infusion bag. The absence of any visible change to a solution upon mixing does not automatically exclude degradation of either or both components. Promethazine is found to be incompatible in morphine sulfate in syringe preparation (3). However, in palliative care settings combinations of drugs in the same syringe for use in a syringe driver is not uncommon (3). In conclusion, mixing of drugs is best avoided. If circumstances warrant mixing, there should be support from published compatibility data (3).
